# A Commentary on “Robot-Assisted Versus Laparoscopic Distal Pancreatectomy in Patients with Resectable Pancreatic Cancer: An International, Retrospective, Cohort Study”

**DOI:** 10.1245/s10434-023-13427-1

**Published:** 2023-04-10

**Authors:** Dingwei Xu, Jie Huang

**Affiliations:** Department of Hepatobiliary and Pancreatic Surgery, The Second Affiliated Hospital of Kunming Medical University, Kunming, China

Dear Editor

We read with interest a recent article by Jeffrey W. Chen et al. published in the *Annals of Surgical Oncology* titled “Robot-Assisted versus Laparoscopic Distal Pancreatectomy in Patients with Resectable Pancreatic Cancer: An International, Retrospective, Cohort Study.” The authors included 542 patients who received minimally invasive distal pancreatectomy in 33 centers across 11 countries (2010–2019) to evaluate the R0-resection, lymph node yield, major complications, conversion rate, and overall survival in robot-assisted distal pancreatectomy (RDP) and laparoscopic distal pancreatectomy (LDP). Overall, although the lymph node yield and conversion rate appeared favorable after RDP, LDP was associated with shorter operative time, fewer major complications, and shorter hospital stay.^1^ However, we would like to discuss some of our concerns.

First, this is a multicenter and retrospective study. Multivariable logistic regression analyses were performed for the two main outcomes of the study, R0 resection and major complications, to determine whether the surgical approach or other variables were significantly associated with both outcomes. However, this study was nonrandomized and nonblinded. Robotic technology offers advantages in overcoming some laparoscopic defects (unstable camera, rigid instruments, and loss of 3D vision)^2^ but may select for patients with tumors close to important blood vessels in the pancreas, specifically those located near the spleen vein and superior mesenteric vein. These patients may be more inclined to choose RDP because it requires major vascular resection and reconstruction. Indeed, Table 2 shows that RDP was associated with a longer operative time (290 versus 240 min, *p* < 0.001) and more vascular resections (7.6% versus 2.7%, *p* = 0.030). No emergency conversions occurred during RDP compared with LDP (0% versus 5.3%, *p* = 0.004), which also indicates that robotic surgery has advantages in the delicate manipulation of blood vessels. If the authors had used propensity scores to better compare the two groups, would the results have been different?

Second, I observed that only centers in which at least 50 minimally invasive distal pancreatectomy (MIDP) procedures were performed for all indications were included. It is unknown whether the participating centers conduct robotic surgery. Is it possible that surgeons in the robotic group were still learning? Multi-institution administrative databases have great utility, but their heterogeneity in data collection and the surgeons’ skills can substantially affect their findings; thus, eliminating biases in surgical skills is difficult. Moreover, the experience levels of each robotic pancreatic surgeon may vary across patients.

Third, I noticed that 10 patients in the RDP and 34 patients in the LDP received preoperative neoadjuvant therapy; however, detailed information on the time between the neoadjuvant therapy and surgery was not provided. It seems that surgery was not performed at a fixed time interval after neoadjuvant therapy, which could affect the results because neoadjuvant therapy exhibited more adhesion in the local area of the pancreas and increased intraoperative bleeding in the case of upfront surgery.^3^ This factor likely affected postoperative complications.

According to the abovementioned concerns, we suggest that RDP is an effective alternative to complex distal pancreatectomy for patients with resectable pancreatic cancer.

## References

[1] Chen Jeffrey W, van Ramshorst Tess ME, Sanne L (2023). Robot-assisted versus laparoscopic distal pancreatectomy in patients with resectable pancreatic cancer: an international, retrospective, cohort study. Ann Surg Oncol..

[2] Gan L, Peng L, Li J (2022). Comparison of the effectiveness and safety of robotic-assisted and laparoscopic in adrenalectomy: a systematic review and meta-analysis. Int J Surg.

[3] Barreto SG, Loveday B, Windsor JA, Pandanaboyana S (2019). Detecting tumour response and predicting resectability after neoadjuvant therapy for borderline resectable and locally advanced pancreatic cancer. ANZ J Surg.

